# Flexible, dual-form nicotine replacement therapy or varenicline in comparison with nicotine patch for smoking cessation: a randomized controlled trial

**DOI:** 10.1186/s12916-016-0626-2

**Published:** 2016-06-07

**Authors:** Heather E. Tulloch, Andrew L. Pipe, Charl Els, Matthew J. Clyde, Robert D. Reid

**Affiliations:** Division of Prevention and Rehabilitation, University of Ottawa Heart Institute, 40 Ruskin Street, Ottawa, ON K1Y 4 W7 Canada; Faculty of Medicine, University of Ottawa, Ottawa, Canada; School of Psychology, University of Ottawa, Ottawa, Canada; Department of Psychiatry, 1E1 Walter Mackenzie Health Sciences Centre, University of Alberta, Edmonton, AB T6G 2R7 Canada

**Keywords:** Smoking cessation, RCT, Efficacy, Intervention, Extended treatment

## Abstract

**Background:**

Extended use of combined pharmacotherapies to treat tobacco dependence may increase smoking abstinence; few studies have examined their effectiveness. The objective of this study was to evaluate smoking abstinence with standard nicotine patch (NRT), extended use of combined formulations of nicotine replacement therapy (NRT+), or varenicline (VR).

**Methods:**

A total of 737 smokers, including those with medical and psychiatric comorbidities, were randomly assigned to one of the above three treatment conditions. The NRT group received 10 weeks of patches (21 mg daily maximum); the NRT+ group received patches (35 mg daily maximum) and gum or inhaler for up to 22 weeks; and the VR group received 1 mg twice daily for up to 24 weeks (22 weeks post target quit date). All participants also received six standardized 15-minute smoking cessation counseling sessions by nurses experienced in tobacco dependence treatment. The primary outcome was carbon monoxide-confirmed continuous abstinence rates (CAR) from weeks 5–52. Secondary outcomes were: CAR from weeks 5–10 and 5–22, and carbon monoxide-confirmed 7-day point prevalence (7PP) at weeks 10, 22, and 52. Adjusted and unadjusted logistic regression analyses were conducted using intention-to-treat procedures.

**Results:**

The CARs for weeks 5–52 were 10.0 %, 12.4 %, and 15.3 % in the NRT, NRT+, and VR groups, respectively; no group differences were observed. Results with 7PP showed that VR was superior to NRT at week 52 (odds ratio (OR), 1.84; 97.5 % Confidence Interval (CI), 1.04–3.26) in the adjusted intention-to-treat analysis. Those in the VR group had higher CAR at weeks 5–22 (OR, 2.01; CI, 1.20–3.36) than those in the NRT group. Results with 7PP revealed that both NRT+ (OR, 1.72; CI, 1.04–2.85) and VR (OR, 1.96; CI, 1.20–3.23) were more effective than NRT at 22 weeks. As compared to NRT monotherapy, NRT+ and VR produced significant increases in CAR for weeks 5–10 (OR, 1.52; CI, 1.00–2.30 and OR, 1.58; CI, 1.04–2.39, respectively); results were similar, but somewhat stronger, when 7PP was used at 10 weeks (OR, 1.57; CI, 1.03–2.41 and OR, 1.79; CI, 1.17–2.73, respectively). All medications were well tolerated, but participants in the VR group experienced more fatigue, digestive symptoms (e.g., nausea, diarrhea), and sleep-related concerns (e.g., abnormal dreams, insomnia), but less dermatologic symptoms than those in the NRT or NRT+ groups. The frequency of serious adverse events did not differ between groups.

**Conclusions:**

Flexible and combination NRT and varenicline enhance success in the early phases of quitting. Varenicline improves abstinence in the medium term; however, there is no clear evidence that either varenicline or flexible, dual-form NRT increase quit rates in the long-term when compared to NRT monotherapy.

**Trial registration:**

ClinicalTrials.gov Identifier: NCT01623505; Retrospectively registered on July 13, 2011

## Background

Despite the well-established consequences [1], smoking remains prevalent worldwide [1–3]. Cessation attempts are common; 40 % of smokers try to quit at least twice annually [4], but only 3–5 % will be successful [5]. Pharmacotherapy and behavioral interventions improve quit rates [6, 7]. Evidence based on indirect comparisons derived from meta-analyses favors combinations of nicotine replacement therapy (NRT) or varenicline [6–8] over NRT monotherapy. To date, most studies have compared standard-dose NRT or varenicline to placebo; only a handful of studies have compared NRT monotherapy to combinations of NRT products or varenicline [6–11], and only one study [12] has included NRT monotherapy, combination NRT, and varenicline in a single trial, with the results proving inconsistent with previous meta-analyses and clinical practice guidelines [7]. Additional comparisons of these treatments are required to provide direct evidence of their relative effectiveness.

Little is known about the efficacy of smoking cessation treatments in populations with significant medical or psychiatric comorbidities [13–15]; these groups are typically excluded from trials. Cardiac patients, for example, who may gain immediate benefits from quitting are often deemed ineligible for participation in smoking cessation research due to concerns of potential adverse events. In fact, a recent review reported that only two randomized controlled trials (RCTs) investigated varenicline prescribed to patients with active cardiovascular disease and 11 studies included patients with a history of cardiovascular disease [16]. Studies examining cessation among those with psychiatric diagnoses are limited by small sample size, have omitted formal diagnostic procedures, or have examined only select psychiatric populations. This is particularly true of trials assessing varenicline – only six RCTs examining psychiatric patients have been published and three included fewer than 50 smokers [17–22]. As rates of smoking are disproportionately higher in those with psychiatric disorders, RCTs including such patients should be a priority. The present trial includes patients with physical and psychiatric comorbidities and it is the first to directly compare NRT and varenicline in smokers with and without psychiatric illness.

The primary objective of this randomized controlled trial was to compare the effectiveness of three cessation treatment strategies: standard-dose monotherapy NRT (NRT); extended duration of combinations of NRT products (NRT+); and extended varenicline (VR). These interventions were chosen as they are first-line treatments for smoking cessation, and the use of flexible and dual-form NRT reflects contemporary clinical practice and the choices of smokers in the real world; the efficacy of this treatment and varenicline compared to standard-dose nicotine replacement therapy requires further testing. We hypothesized that NRT+ and/or VR would each be superior to standard-dose NRT in achieving smoking cessation.

## Methods

### Study design

Written consent forms and study procedures were approved by the Ottawa Health Sciences Network Research Ethics Board, and the trial was registered on ClinicalTrials.gov (identifier# NCT01623505). All participants provided voluntary written informed consent. This parallel, three-group randomized controlled trial was conducted at a single center (Ottawa Heart Institute) between June 2010 and July 2014. Participants were randomized to one of the three treatment conditions: NRT, NRT+, or VR. Six standardized 15-minute smoking cessation counseling sessions by nurses experienced in tobacco dependence treatment were provided to all participants; sessions were offered in-person on an individual basis.

### Eligibility and screening

Participants were recruited by advertising (i.e., radio, local newspaper, and posters), from those presenting to the Quit Smoking Program at our institution, and from referrals by local physicians. Interested smokers contacted the study coordinator by phone or in-person and were screened for eligibility. A baseline visit was scheduled at which eligibility was reconfirmed. Eligible participants were 18 years or older, smoked ≥ 10 cigarettes per day, and were willing to make a quit attempt in the next 2–4 weeks. Participants were initially excluded if they had used any of the study medications in the previous 6 months; this proved to be overly restrictive and unnecessary due to the short medication wash out period and, subsequently, was revised. Exclusion criteria included the use of NRT or varenicline for more than 72 consecutive hours in the past month; the presence of contra-indications to the use of study medications; serious cardiac arrhythmias or a myocardial infarction or cerebral vascular accident within the previous 10 days; severe or unstable angina pectoris; end-stage renal disease or use of cimetidine; alcohol or substance abuse in the previous 3 months; unstable psychiatric symptoms precluding informed consent (i.e., active, untreated psychosis or suicidality); refusal to be randomized; unable to attend follow-up appointments; and an inability to understand English or French. Women were excluded if pregnant, lactating, or likely to become pregnant in the next year. No more than one person from the same household was permitted to participate.

### Study procedures

Participants attended a baseline assessment, which included a medical and psychiatric assessment, and completed questionnaires assessing demographics, smoking history, and nicotine dependence. Women provided a urine sample to test for pregnancy. After eligibility was confirmed by one of the principal investigators (HT, AP), participants were randomized to receive NRT, NRT+, or VR using a computer-generated block randomization schedule by a statistical consultant not involved in the trial; block sizes varied from 6–12. Randomization was stratified by psychiatric status (yes/no). Participants were seen on eight occasions including a baseline assessment and first counseling session at week 0, and follow-ups at 1, 3, 5, 8, 10, 22, and 52 weeks post-target quit date. Counseling and medication distribution occurred at the first six visits; a 5-week supply was provided at baseline and week 5 to prevent missed doses if a participant was absent at a follow-up appointment. Participants selected their target quit date; those using nicotine replacement therapies set this date 1–14 days from the baseline assessment, and participants using varenicline set a date on day 8–14 after baseline. At each appointment, withdrawal symptoms, smoking status, medication usage, and adverse events were assessed. Exhaled carbon monoxide (CO) levels were determined at weeks 5, 10, 22, and 52. Study nurses collected outcome data during the treatment phase, and the research coordinator, who was blind to treatment condition, collected follow-up data (week 22 and 52). Further details regarding study design, randomization procedures, counseling content, resources and staff training have been described previously [23].

### Pharmacological interventions

All medications were purchased with study funds from the Ottawa Heart Institute Pharmacy, and were provided to participants on an open label basis. The research coordinator collecting follow-up data at weeks 22 and 52 was blind to treatment condition.

#### NRT group

A 10-week supply of Nicoderm® patches was provided to participants. The first patch was applied on the target quit date. Initial dosing was determined by the daily average of cigarettes smoked: ≥ 20 cigarettes/day received 21 mg/day for 6 weeks, 14 mg/day for 2 weeks, and 7 mg/day for 2 weeks, while those smoking less were prescribed 14 mg/day for 6 weeks and 7 mg/day for 4 weeks.

#### NRT+ group

Treatment for the NRT+ group was similar to that in the NRT group; however, the fixed-dose strategy and 10-week duration of therapy was not applied. Instead, participants were encouraged to address withdrawal symptoms by titrating their NRT use up to a daily maximum of 35 mg via patches and to use Nicorette® gum or inhalers ad libitum. The Minnesota Nicotine Withdrawal Scale [24] was used to assist participants and staff to titrate dosing at each visit; scores ≥ 2 on any item signaled a need to increase the dosage. If interested and it was recommended by the study nurse or physician, participants could continue to receive treatment for up to 22 weeks.

#### VR group

Participants assigned to the VR group began the medication (i.e., Champix) at the baseline assessment. The dosage was 0.5 mg once daily for 3 days, increasing to 0.5 mg twice daily for days 4–7, followed by a maintenance dose of 1 mg twice daily for 11 weeks. If interested and it was recommended by the study nurse or physician, participants could receive a second 12-week supply of varenicline at the week 10 counseling session. Thus, treatment could be provided for up to 24 weeks (i.e., 22 weeks post-target quit date).

### Measures

At baseline, age, sex, ethnicity, marital and employment status, education level, income (Canadian dollars), number of smokers in the household, the age of onset of smoking, number of years as a daily smoker, number of previous quit attempts, number of cigarettes smoked per day, and motivation and confidence to quit (1–10 scale) were collected from each smoker. A self-reported medical history was obtained by the study nurse or physician and medication use documented. The presence of a psychiatric diagnosis was determined using the Mini International Neuropsychiatric Interview 6.0.0. (MINI) [25]. Nicotine dependence was assessed with the 6-item Fagerstrom Test for Nicotine Dependence [26]; scores ≥ 6 indicate high dependence. During the treatment phase, the Minnesota Nicotine Withdrawal Scale was used to assess withdrawal symptoms during the previous 24 hours [24]. Adherence to the study medication was assessed by dividing the amount used by the amount dispensed. Adverse events were noted at each visit, and the study nurse (treatment phase) or study coordinator (follow-up phase) inquired about changes in the participants’ health, medications, and any recent hospitalizations.

The primary efficacy end point was the CO-confirmed continuous abstinence rate (CAR) during weeks 5–52. As per the Russell Standard [27], a participant was considered abstinent if he or she smoked five cigarettes or less during that period and had an exhaled CO level of ≤ 9 ppm at the 52-week visit. Participants were considered smokers if a visit was missed or dropped out from the study. Secondary outcomes included CO-confirmed CAR from 5–10 and 5–22 weeks post-target quit date and CO-confirmed 7-day point prevalence abstinence (7PP) at weeks 5, 10, 22, and 52 (i.e., smoked no cigarettes, even a puff, in the last 7 days, confirmed with CO exhalation).

### Statistical analyses

The primary endpoint used to determine sample size was the CAR measured from weeks 5–52. The sample size was calculated based on the assumption that the proportion to quit in the NRT group would be 0.20 compared to 0.30 in the VR group and 0.35 in the NRT+ group [7]. A priori analyses indicated that 854 participants were required to detect differences in abstinence rates between the groups; we planned to increase this sample size to 1068 to account for attrition (20 %). The primary analysis was based on an intent-to-treat approach but, as per the Russell Standard [27], was extended such that participants who had missing outcome data were considered smokers [27]. Participants that died or were no longer reachable (i.e., moved, phone not in service) were removed from the analysis [27]. An unadjusted logistic regression model that included treatment group as the independent variable (NRT as reference group) was fit to the smoking status (abstinent or not) from weeks 5–52 for the primary analysis. These analyses were repeated with the secondary outcomes (CAR from weeks 5–10 and 5–22, as well as 7PP collected at weeks 10, 22, and 52). An adjusted logistic regression model was conducted including baseline variables that were potential univariate predictors of cessation outcome (conservative univariate *P* value set at 0.15). As the assumption that missing data is equivalent to smoking may bias the treatment effect analysis, we also conducted a sensitivity analysis by assuming an imperfect relationship between missing and smoking status. As per Hedeker et al. [28], we assumed different odds ratios for this relationship and compared these to the intention-to-treat analysis. In addition, we conducted the logistic regression analyses with responders only. Finally, exploratory logistic regression analyses were conducted to investigate the effect of medication extension on smoking status. For all analyses of smoking-cessation outcomes, an alpha level of 0.025 was used to adjust for multiple comparisons. As such, odds ratios (OR) with 97.5 % confidence intervals (CI) are reported throughout. χ^2^ analyses with Bonferroni-adjusted post hoc tests (alpha level 0.05) were performed to examine the relationships between adverse events and treatment condition.

## Results

### Enrollment and follow-up

**Fig. 1:**
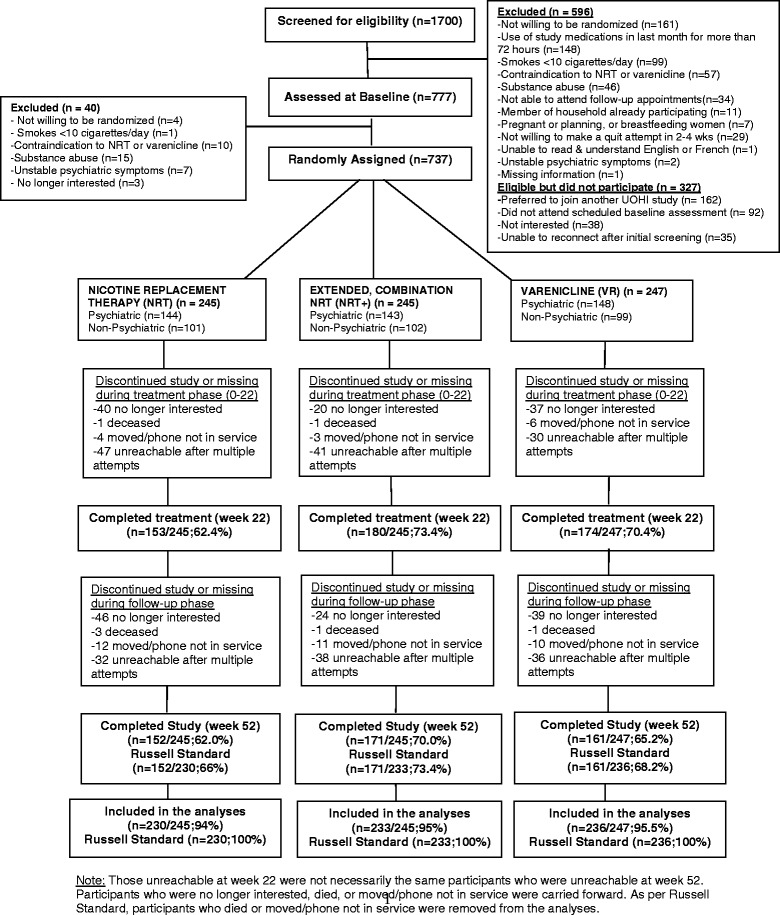
Recruitment and retention for the FLEX trial

From 1700 potentially eligible participants, 737 were randomly assigned to treatment: NRT (*n* = 245), NRT+ (*n* = 245), and VR (*n* = 247). With 245 participants per group and an alpha of 0.025 to account for multiple comparisons, we had 63 % power to detect a 10 % improvement in quit rates between the NRT and VR groups and 93 % power to detect a 15 % improvement in quit rates between NRT and NRT+. Follow-up rates at the end of treatment (week 22) were 62.4 % (*n* = 153) in the NRT group, 73.4 % (*n* = 180) in the NRT+ group, and 70.4 % (*n* = 174) in the VR group. At study completion, follow-up rates were 62.0 % (*n* = 152), 70.2 % (*n* = 172), and 65.2 % (*n* = 161), respectively; these rates were 66.0 % (*n* = 152), 73.4 % (*n* = 171), and 68.2 % (*n* = 161), respectively, using the Russell Standard excluding 15, 12, and 11 participants, respectively, due to death or moving/phone out of service (Fig. 1).

### Demographic, medical and smoking profile

Sample characteristics were similar between the treatment groups at baseline (see Table 1 and previous publication [23]). Participants had a mean (M) age of 48.6 (standard deviation (SD), 10.8; Median (Mdn), 50; interquartile range (IQR), 42–56) years, 53.6 % (*n* = 395) were male, 91.8 % (*n* = 669) were white, had 14 (SD, 3.0; Mdn, 14; IQR, 12–16) years of education and 52.0 % (*n* = 381) were employed full-time. Most participants (82.9 %; *n* = 611) had at least one medical condition; the most prevalent conditions were chronic pain (39.1 %; *n* = 286), respiratory illness (34.7 %; *n* = 255), and arthritis (31.2 %; *n* = 229). Many participants (59.0 %; *n* = 435) met criteria for a lifetime psychiatric diagnosis; major depressive disorder (64.6 %; *n* = 281) and anxiety disorders (21.4 %; *n* = 93) were most prevalent. Smoking had begun during adolescence (M age, 14.5; SD, 4.0 years; Mdn, 20; IQR, 15–25), and participants smoked for 31 (SD, 11.7) years on average consuming 23.2 (SD, 10.8; Mdn, 32; IQR, 23–32) cigarettes per day. Participants tended to live with one smoker (M, 1.04; SD, 1.02); 66.2 % (*n* = 475) lived with one or more smokers. High nicotine dependence (M, 6.1; SD, 2.2; Mdn, 6; IQR, 5–8) and confidence (M, 7.38; SD, 2.16; Mdn, 8; IQR, 6–8) and motivation (M, 8.69; SD, 1.47; Mdn, 9; IQR, 8–9) to quit were evident at the baseline assessment.

**Table 1:** Baseline demographic information

*n* (%)	Overall	NRT	NRT+	VR
Age (M, SD)	48.61 (10.8)	48.13 (11.1)	48.57 (10.5)	49.11 (10.8)
Sex				
Male	395 (53.6)	137 (55.9)	131 (53.5)	127 (51.4)
Female	342 (46.6)	108 (44.1)	114 (46.5)	120 (48.6)
Marital status				
Married/common law	340 (46.3)	120 (49.0)	107 (43.9)	113 (46.3)
Divorced/separated/widowed	232 (31.7)	69 (28.1)	79 (32.3)	84 (34.4)
Single/never married	161 (22.0)	56 (22.9)	58 (23.8)	47 (29.2)
Years of education (M, SD)	14.13 (3.0)	13.97 (2.7)	14.19 (3.0)	14.21 (3.2)
Employment status				
Full/part time	449 (61.3)	157 (64.4)	145 (59.5)	147 (60.1)
Homemaker/retired/unemployed	158 (21.6)	49 (20.0)	51 (20.8)	58 (23.6)
Disability leave	126 (17.1)	38 (15.6)	48 (19.7)	40 (16.3)
Annual household income (Canadian dollars)				
19,999 or less	162 (22.7)	50 (20.7)	61 (25.8)	51 (21.6)
20,000–39,999	147 (20.6)	51 (34.7)	47 (19.9)	49 (20.8)
40,000–69,999	216 (30.3)	76 (31.5)	67 (28.4)	73 (30.9)
70,000 or more	188 (26.4)	64 (26.6)	61 (26.8)	63 (26.7)
Lifetime psychiatric diagnosis (%)	435 (59.0)	144 (58.5)	143 (58.4)	148 (58.9)
Smoking characteristics (M, SD)				
Cigarettes smoked per day	23.2 (10.8)	22.4 (11.3)	24.0 (10.9)	23.3 (10.1)
Cumulative years smoked	31.0 (11.7)	30.5 (12.2)	30.9 (11.3)	31.7 (11.5)
Fagerstrom test of nicotine dependence	6.1 (2.2)	6.0 (2.2)	6.3 (2.2)	6.1 (2.3)
Number of previous quit attempts	4.6 (5.4)	4.3 (4.5)	4.2 (4.5)	5.2 (6.7)
Motivation to quit^a^	8.7 (1.5)	8.7 (1.4)	8.7 (1.6)	8.7 (1.4)
Confidence to quit^a^	7.4 (2.2)	7.3 (2.2)	7.4 (2.1)	7.4 (2.2)
Number of other smokers in the household	1.0 (1.0)	1.1 (0.9)	1.0 (0.9)	1.1 (1.2)

M, mean; SD, standard deviation; NRT, standard dose, nicotine replacement therapy; NRT+, flexible, dual-form nicotine replacement therapy; VR, varenicline

^a^Motivation and confidence to quit were reported on 10-point scales where 0 = not at all confident/motivated to and 10 = completely confident. Please note that this table is an adapted version of a previously published table [23]

### Intervention

The median medication dose at week 5 was 21 mg, 21 mg, and 1 mg for NRT, NRT+, and VR, respectively. At week 10, reductions were noted for the NRT group (Mdn, 7 mg), but remained the same for the NRT+ and VR groups. The maximum average daily dosage of nicotine patches used by NRT+ participants was 24.4 mg daily (SD, 6.7 mg; range, 14–35 mg). Most participants (80.4 %) in the NRT+ group chose to use inhalers over gum. Adherence rates were similar across groups (*P* = 0.08): 84.3 % (*n* = 194), 79.7 % (*n* = 198), and 78.9 % (*n* = 200), respectively.

### Smoking abstinence

Table 2 displays the abstinence rates by treatment condition as well as results from the unadjusted and adjusted logistic regression models. The CARs for weeks 5–52 were 10.0 % (*n* = 24), 12.4 % (*n* = 30), and 15.3 % (*n* = 37) in the NRT, NRT+, and VR groups, respectively; they were not significantly different between groups (*P* > 0.025). Results with 7PP showed that VR was superior to NRT at week 52 (OR, 1.84; CI, 1.04–3.26) in the adjusted intention-to-treat analysis. Those in the VR group had higher CAR at weeks 5–22 (OR, 2.01; CI, 1.20–3.36) than those in the NRT group. Results with 7PP revealed that both NRT+ (OR, 1.72; CI, 1.04–2.85) and VR (OR, 1.96; CI, 1.20–3.23) were more effective than NRT at 22 weeks. As compared to NRT monotherapy, NRT+ and VR produced significant increases in CAR for weeks 5–10 (OR, 1.52; CI, 1.00–2.30 and OR, 1.58; CI, 1.04–2.39, respectively); results were similar, but somewhat stronger, when 7PP was used at 10 weeks (OR, 1.57; CI, 1.03–2.41 and OR, 1.79; CI, 1.17–2.73, respectively). Overall, the logistic regression model adjusting for predictors variables (i.e., marital status, income, nicotine dependence, and motivation) strengthened the observed results (Table 2). Results from the sensitivity analysis were not different from the primary analysis (i.e., intention-to-treat approach with missing data coded as smokers). In comparisons between NRT and NRT+, analysis including responders only produced weaker odds ratios and below significant levels for the NRT+ group at weeks 10 and 22. Responder-only analyses including VR were consistent with the intention-to-treat analyses.

**Table 2:** Abstinence rates and odds ratios by treatment condition

	NRT	NRT+	VR
Primary outcome: continuous abstinence at week 5–52			
Abstinence, *n* (%)	23 (10)	29 (12.4)	36 (15.3)
OR (97.5 % CI)	REF	1.28 (0.67–2.43)	1.62 (0.87–3.01)
Unadjusted			
Adjusted	REF	1.34 (0.67–2.70)	1.84 (0.94–3.58)
Secondary outcome: 7-day point prevalence abstinence at week 52			
*n* (%)	34 (14.8)	43 (18.5)	51 (21.7)
Unadjusted	REF	1.31 (0.76–2.25)	1.60 (0.94–2.70)
Adjusted	REF	1.31 (0.79–2.37)	1.84 (1.04–3.26)*
Secondary outcome: continuous abstinence at week 5–22			
*n* (%)	38 (15.8)	59 (24.5)	65 (27.1)
Unadjusted	REF	1.51 (0.88–2.57)	2.01 (1.20–3.36)**
Adjusted	REF	1.67 (0.95–2.94)	2.18 (1.25–3.80)**
Secondary outcome: 7-day point prevalence at week 22			
*n* (%)	38 (15.8)	59 (24.5)	65 (27.1)
Unadjusted	REF	1.72 (1.04–2.85)*	1.96 (1.20–3.23)**
Adjusted	REF	1.87 (1.09–3.20)*	2.09 (1.22–3.57)**
Secondary outcome: continuous abstinence week 5–10			
*n* (%)	72 (29.4)	94 (38.7)	98 (39.8)
Unadjusted	REF	1.52 (1.00–2.30)	1.58 (1.04–2.39)*
Adjusted	REF	1.62 (1.03–2.56)*	1.66 (1.04–2.63)*
Secondary outcome: 7-day point prevalence at week 10			
*n* (%)	65 (26.5)	88 (36.2)	97 (39.4)
Unadjusted	REF	1.57 (1.03–2.41)*	1.79 (1.17–2.73)**
Adjusted	REF	1.65 (1.04–2.61)*	1.89 (1.19–3.01)**

REF, reference group; OR, odds ration; 97.5 % CI, 97.5 % confidence interval; NRT, standard dose, nicotine replacement therapy; NRT+, flexible, dual-form nicotine replacement therapy; VR, varenicline

Adjusted for marital status, income, nicotine dependence, psychiatric status and motivation to quit. *P < 0.025; **P < 0.01

Over half of the participants in the NRT+ (64.5 %; *n* = 158) and VR (53.4 %; *n* = 132) groups extended treatment beyond the initial intervention period (i.e., 10 weeks). Those who chose to extend were more likely to be older (*P* = 0.007) and smoke-free at 10 weeks (*P* < 0.001); no group differences were found for any of the remaining baseline demographic and smoking-related variables. In order to evaluate the effects of the extended treatment (i.e., the use of study medications past 10 weeks) above and beyond the type or combination of medication, we conducted an exploratory logistic regression analysis comparing five conditions: NRT alone (reference group, *n* = 244), NRT+ without extension (*n* = 85), NRT+ with extension (*n* = 158), VR without extension (*n* = 91), and VR with extension (*n* = 132). Results showed that CAR from 5 to 22 weeks in the non-extension arms were not significantly different from the 10-week NRT monotherapy; quit rates were 14.3 % (*n* = 34) for NRT, 9.4 % (*n* = 8) for non-extended NRT+, 25.3 % (*n* = 40) for extended NRT+, 21.9 % (*n* = 20) for non-extended VR, and 30.3 % (*n* = 40) for extended VR. Participants in the extended conditions, however, had more success in their quit attempts from weeks 5–22 as compared to NRT monotherapy (OR, 2.05; CI, 1.17–3.61 for extended NRT+ and OR, 2.69; CI, 1.51–4.79 for extended VR). Participants who extended their varenicline use were more likely to be continuously abstinent from weeks 5–52 (9.4 %, *n* = 23, NRT; 12.1 %, *n* = 11, non-extended VR; 18.9 %, *n* = 25, extended VR; OR, 2.14; CI, 0.92–4.97) as compared to NRT. Results were similar, but slightly stronger, in the adjusted analyses controlling for marital status, income, nicotine dependence, psychiatric status and motivation to quit.

### Adverse events

Table 3 presents the adverse events reported by participants by treatment condition. Participants in the VR group experienced more fatigue, digestive symptoms (e.g., nausea, diarrhea), and sleep-related concerns (e.g., abnormal dreams, insomnia) than those in the NRT or NRT+ groups. Those in the VR group were less likely to have dermatologic symptoms (e.g., skin rash or irritation). Adverse events resulting in treatment discontinuation by the qualified investigator were not significantly different between the groups (1.6 % (*n* = 4) NRT group; 2 % (*n* = 5) NRT+ group; and 2 % (*n* = 5) VR group; *P* = 0.93). The frequency of serious adverse events did not differ between groups (3.7 % (*n* = 9) in the NRT group; 2.4 % (*n* = 6) in the NRT+ group; and 3.2 % (*n* = 8) in the VR group; *P* = 0.073); only three were deemed to be possibly related to the study medication (VR, 2; NRT+, 1).

**Table 3:** Adverse events by treatment condition

Adverse event	Treatment group *n* (%)			
	NRT	NRT+	VR	*P*
Cardiovascular (e.g., palpitations, tachycardia, chest pain)	5 (2.0)	3 (1.2)	3 (1.2)	0.687
Digestive (e.g., indigestion, nausea, diarrhea, constipation, flatulence)	48 (19.6)	64 (26.1)	139 (56.3)	<0.001^a^
Muscular (e.g., hypertonia, join, neck or jaw pain)	7 (2.9)	5 (2.0)	4 (1.6)	0.632
Nervous system (e.g., dizziness, light headedness, tingling fingers)	24 (9.8)	27 (11.0)	31 (12.6)	0.622
Psychiatric (e.g., anxious, disturbed concentration, suicidal ideation)	12 (4.9)	9 (3.7)	18 (7.3)	0.267
Sleep (e.g., abnormal dreams, insomnia, sleep disturbance)	92 (37.6)	115 (46.9)	149 (60.3)	<0.001^a^
Fatigue (e.g., drowsy, lethargic)	9 (3.7)	19 (7.8)	43 (17.4)	<0.001^a^
Metabolic (e.g., increased appetite, taste perversion)	13 (5.3)	14 (5.7)	23 (9.3)	0.151
Respiratory (e.g., coughing, congestion, shortness of breath)	6 (2.4)	9 (3.7)	10 (4.0)	0.592
Skin (e.g., rash, itchiness, dry skin, redness)	94 (38.4)	81 (33.1)	12 (4.9)	<0.001^a^
Other (e.g., eyes difficult to focus, hot flashes, low sex drive)	21 (8.6)	19 (7.8)	48 (19.4)	<0.001^a^

NRT, standard dose, nicotine replacement therapy; NRT+, flexible, dual-form nicotine replacement therapy; VR, varenicline

^a^Statistically significant

## Discussion

Among smokers with and without medical and psychiatric diagnoses, no differences in CAR from weeks 5–52 were observed between the treatment groups; VR, however, significantly improved the odds of quitting when 7DPP was the outcome variable in the adjusted model at one year (*P* = 0.019). Both NRT+ and VR produced statistically significant increases in CAR from weeks 5–10; and VR resulted in greater abstinence rates from weeks 5–22 when compared to NRT monotherapy. Using 7PP, NRT+ was also more effective than NRT at 22. Of note, the analysis with responders only found insignificant results for all time points in the comparisons between NRT and NRT+, likely indicating a possible overestimation of the effectiveness of the NRT+ intervention. Nonetheless, the NRT+ quit rates were 10 % higher at 10 weeks and 8.7 % higher at 22 weeks, which are clinically significant. Our results suggest that both varenicline and extended and combination NRT enhance success in the early phases of quitting; varenicline improves abstinence in the medium-term and that there is no clear evidence that varenicline or combined NRT increase quit rates in the long-term in comparison to NRT monotherapy. Similar results were reported in a recent study with these treatment groups, albeit with reduced treatment duration (i.e., 12 weeks) [12].

Using the secondary outcome of 7DPP, only VR produced improved quit rates in comparison to NRT at 1 year. Previous investigations have demonstrated the safety of this medication [17, 29–32]. In our sample, differences in the frequency of adverse events were observed: VR use was related to higher levels of fatigue and sleep and digestive problems, and fewer reports of skin problems as compared to the groups using nicotine replacement therapies. The experience of these symptoms, however, did not translate into an unbalanced distribution of treatment discontinuation by condition. Further, no differences in psychiatric adverse events were detected, including between the psychiatric and non-psychiatric groups in our sample. We argue that smokers with psychiatric illness may be offered treatment options similar to those in the general population; their choice should not be restricted by the presence of a psychiatric diagnosis. In addition, the cost of varenicline is at least half that of combination NRT, a factor that is important to many smokers attempting to quit.

Our quit rates are similar but slightly lower than those reported in previous studies [9, 10, 17, 33] and a recent meta-analysis reporting abstinence at 6 months or more [6]. Lower rates of cessation may reflect the current population of smokers: those with higher nicotine dependence and significant comorbidities who have greater difficulty quitting [34, 35]. The sociodemographic and medical profile of our participants provides evidence of higher nicotine dependence and significant comorbidities among current smokers. Our sample had multiple medical conditions (82.9 % (*n* = 611) reported at least one; 12.6 % (*n* = 93) reported > 5; 59.0 % (*n* = 435) had a lifetime psychiatric diagnosis; 64.3 % (*n* = 455) fell in the high nicotine dependence category (i.e., > 6 in the Fagerstrom Test for Nicotine Dependence test); and 22.7 % (*n* = 162) live in poverty (i.e., annual income < $20,000). As we attempted to provide an intervention that might be amenable to real-world settings, we provided fewer counseling sessions than typically provided in efficacy trials [33], which may be another explanation for our lower quit rates.

Adverse events reported in our study appear higher than those reported in previous investigations [17, 33]. There are two potential explanations: first, adverse events were categorized and summed in our study, while other studies often count each symptom occurrence independently. For example, sleep disturbances (e.g., abnormal dreams, insomnia, and hypersomnia) were tallied in our study, but presented individually in previous studies. Second, participants in our trial were not blind to treatment condition nor was a placebo control group established; this may have contributed to participants seeking, detecting, perceiving, and/or interpreting symptoms more frequently and assigning causality to the study medication leading to increased reporting of adverse events. Nonetheless, these symptoms were well-tolerated as treatment discontinuation was limited and did not differ by condition.

The major strengths of this trial were its randomized design, validated outcomes, and the particular inclusion of smokers typically excluded from cessation studies. It is one of few studies to compare monotherapy NRT to extended, combined NRT or extended varenicline [6, 8, 11, 12, 36, 37], and the second to include these treatments in a single trial. Our results add to the literature regarding more effective use of smoking cessation pharmacotherapy; the long-term results of flexible, dual-form NRT and varenicline are less clear and put into question the early indicators derived from meta-analyses and clinical experience.

Our study is not without limitations. Although we employed conservative estimates for sample size calculations, it remains possible that clinically important differences in quit rates between the treatment groups were not detected due to an inadequate sample size which fell below our planned levels (i.e., 737 participants enrolled versus 854 required to detect differences and 1068 planned to recruit to account for attrition); this is particularly true for the comparison between VR and NRT+ (63 % power). The generalizability of these three treatment methods to a broader population of smokers with comorbid conditions is unknown. For example, we are limited by our sample being comprised mainly of white participants. Unequal sample sizes were evident in the exploratory analyses investigating length of treatment. Further, this analysis no longer included the balancing of potential confounders because patients self-selected whether to extend treatment or not; however, no differences were detected between groups on any baseline demographic or smoking-related variables, and we controlled for variables which significantly predicted outcomes in the analysis. A randomized controlled trial including these five treatment conditions would solidify the findings reported herein. Such a trial would clarify if the treatment extension or therapy dose produced the enhanced success in quitting. At present, however, our study design was limited by the use of both increased medication dosage and duration of treatment in the NRT+ group (≤35 mg, maximum 22 weeks) than in the NRT group (≤21 mg, maximum 10 weeks); increases in one or the other would have allowed for more definitive conclusions regarding treatment efficacy. Nonetheless, similar results to those reported herein have been reported by recent trials which kept duration constant [12] or restricted treatment to single-form only [37].

We were also limited by the lack of a placebo condition. The study staff was aware of treatment allocation during the treatment phase; there may have been reporting bias in favor of one treatment versus another or in the expectation of adverse events. This effect was reduced, however, by the fact that multiple nurses were involved in the assessment and staff collecting follow-up data at weeks 22 and 52 were blind to treatment condition. There may have been a reporting bias of adverse events by participants. Future research might record participants’ treatment preference prior to randomization to better understand the effects of preference on efficacy outcomes and adverse advent reporting. Our study may have been strengthened by identifying potential confounders for the adjusted analysis a priori. Although we made concerted efforts to retain and follow all randomly assigned participants throughout the 52-week trial, high attrition rates were noted across all treatment groups, and missing data could have affected outcomes. The assumption that all drop-outs have resumed smoking could underestimate abstinence rates; however, empirical evidence from smoking cessation studies suggests that drop-outs tend to relapse to smoking [27] and our sensitivity analysis did not indicate differences from the intention-to-treat analysis. The analyses with responders only, however, did reveal that results may be overestimated with regards to the extended and combined NRT group.

## Conclusion

Our trial suggests that, in comparison to a standard nicotine patch, flexible and dual-form NRT and varenicline enhance cessation in the short-term but, unfortunately, the effect appears to wane over time. Although both NRT+ and VR led to increased odds of quitting from 5 to 10 weeks and VR produced improved cessation rates from 5 to 22 weeks, only clinically significant differences between the NRT and VR groups (5.3 %) were observed at 1 year. The results presented herein, therefore, do not fully support the use of flexible, dual-form nicotine replacement therapies or varenicline rather than standard nicotine patch for smoking cessation maintenance. Future research is needed to clarify the effectiveness of these treatments; trials that investigate both the dose and duration of each treatment condition are needed.

## Abbreviations

NRT, nicotine replacement therapy; NRT+, flexible and dual-form nicotine replacement therapy; VR, varenicline; CAR, carbon monoxide confirmed, continuous abstinence rate; 7PP, carbon monoxide confirmed, 7 day point prevalence abstinence; OR, odds ratio; CI, confidence interval; RCT, randomized controlled trial; MINI, Mini International Neuropsychiatric Interview; Mdn, median; IQR, interquartile range; FTND, Fagerstrom test of nicotine dependence; CO, carbon monoxide
